# Adenosine monophosphate activated protein kinase contributes to skeletal muscle health through the control of mitochondrial function

**DOI:** 10.3389/fphar.2022.947387

**Published:** 2022-10-20

**Authors:** Yan Yan, Ming Li, Jie Lin, Yanan Ji, Kexin Wang, Dajun Yan, Yuntian Shen, Wei Wang, Zhongwei Huang, Haiyan Jiang, Hualin Sun, Lei Qi

**Affiliations:** ^1^ Department of Emergency Medicine, Affiliated Hospital of Nantong University, Nantong, China; ^2^ Key Laboratory of Neuroregeneration of Jiangsu and Ministry of Education, Co-Innovation Center of Neuroregeneration, NMPA Key Laboratory for Research and Evaluation of Tissue Engineering Technology Products, Jiangsu Clinical Medicine Center of Tissue Engineering and Nerve Injury Repair, Nantong University, Nantong, China; ^3^ Department of Laboratory Medicine, Binhai County People’s Hospital Affiliated to Kangda College of Nanjing Medical University, Yancheng, China; ^4^ Department of Infectious Disease, Affiliated Hospital of Nantong University, Nantong, China; ^5^ Department of Pathology, Affiliated Hospital of Nantong University, Medical School of Nantong University, Nantong, China

**Keywords:** AMPK, mitochondria, skeletal muscle, muscle atrophy, muscle regeneration

## Abstract

Skeletal muscle is one of the largest organs in the body and the largest protein repository. Mitochondria are the main energy-producing organelles in cells and play an important role in skeletal muscle health and function. They participate in several biological processes related to skeletal muscle metabolism, growth, and regeneration. Adenosine monophosphate-activated protein kinase (AMPK) is a metabolic sensor and regulator of systemic energy balance. AMPK is involved in the control of energy metabolism by regulating many downstream targets. In this review, we propose that AMPK directly controls several facets of mitochondrial function, which in turn controls skeletal muscle metabolism and health. This review is divided into four parts. First, we summarize the properties of AMPK signal transduction and its upstream activators. Second, we discuss the role of mitochondria in myogenesis, muscle atrophy, regeneration post-injury of skeletal muscle cells. Third, we elaborate the effects of AMPK on mitochondrial biogenesis, fusion, fission and mitochondrial autophagy, and discuss how AMPK regulates the metabolism of skeletal muscle by regulating mitochondrial function. Finally, we discuss the effects of AMPK activators on muscle disease status. This review thus represents a foundation for understanding this biological process of mitochondrial dynamics regulated by AMPK in the metabolism of skeletal muscle. A better understanding of the role of AMPK on mitochondrial dynamic is essential to improve mitochondrial function, and hence promote skeletal muscle health and function.

## 1 Introduction

Skeletal muscle accounts for 40–50% of lean body mass, making it one of the largest organs in the body and the largest protein respository (Sartori et al., 2021). It plays an important role in posture maintenance, exercise tolerance, temperature regulation, and systemic metabolism (Leduc-Gaudet et al., 2021; Wang et al., 2022a). Reduced and discontinued use, cancer cachexia, nerve injury, diabetes, or inflammation can cause skeletal muscle atrophy (Sun et al., 2014; You and Chen, 2021). Atrophy increases the incidence of pathological fractures, deterioration of organ function, and hospitalization rate, which greatly reduces patients’ quality of life and may even be life-threatening, muscle mass is also a predictor of mortality (Andres-Mateos et al., 2013; Gu, 2021). Therefore, maintaining constant muscle mass and physiological function is important for overall health (Andres-Mateos et al., 2013; Baskin et al., 2015). Skeletal muscle consumes much energy compared to other organ systems, and are thus rich in mitochondria. Mitochondria are critical for regulating skeletal muscle metabolism due to their diverse functions such as energy production, calcium homeostasis, free radical production, triggering/regulating cell death, and the protein synthesis [Reviewed in (Hood et al., 2019)]. Therefore, maintaining the integrity of mitochondrial structure and function is important for muscle health.

Mitochondria are cellular organelles that are covered by distinct outer and inner membranes. They are the main organelles for intracellular energy production through oxidative phosphorylation (OXPHOS) (Nunnari and Suomalainen, 2012; Andrieux et al., 2021). Mitochondria are semi-autonomous organelles that have their own DNA (mtDNA), which can self-replicate under nuclear coordination and encodes a variety of subunits of electron transport chain complexes I, III, IV, and V [Reviewed in (Gustafsson et al., 2016)]. Mitochondria are highly dynamic organelles that undergo processes such as genesis, fusion, division, transportation, and autophagy with the change of cell state. These dynamic mitochondrial biological behaviors are called mitochondrial dynamics, which are essential for maintaining mitochondrial function and structure (Mishra and Chan, 2016; Heine and Hood, 2020).

Mitochondria are involved several of physiological processes including apoptosis, cell chemotaxis, autophagy, oxidative stress, signal transduction, innate immunity, calcium homeostasis, and stem cell reprogramming [Reviewed in (Deshwal et al., 2020)]. Mitochondria form a complex and interconnected cellular network structure, maintaining cell energy homeostasis through the coordination of biogenesis, dynamic fission, fusion, and autophagy (Drake et al., 2021). When cells carry out various biological activities, adenosine triphosphate (ATP) is hydrolyzed to adenosine diphosphate (ADP) or adenosine monophosphate (AMP), which liberates free energy (Ruprecht et al., 2019). When the level of intracellular ATP decreases, the cells attempt to restore the ATP level and maintain energy supply. Eukaryotes have a highly-evolved energy supply system and can regulate their metabolism according to the availability of nutrition. A key player of this system is adenosine monophosphate-activated protein kinase (AMPK) (Herzig and Shaw, 2018).

AMPK is a cellular energy sensor and one of the cellular regulatory systems to ensure that the production and consumption of ATP in the cells remain balanced (Hardie, 2018; Gonzalez et al., 2020). AMPK is activated in response to sensing increased levels of intracellular AMP and ADP, thereby promoting ATP synthesis (Carling, 2017). AMPK can also regulate mitochondrial function through multiple molecular pathways including peroxisome proliferator-activated receptor gamma coactivator-1 alpha (PGC-1a) and sirtuin 1 (SIRT1) (Chang, 2019). AMPK influences mitochondrial processes such as biogenesis, autophagy, fission, and fusion (Hardie et al., 2012; Drake et al., 2021). In view of the important role of mitochondria in skeletal muscle tissue and the regulatory role of AMPK in mitochondrial biological processes, we hypothesize that AMPK plays an important role in skeletal muscle.

There are some studies regarding AMPK’s control of mitochondrial function and the role of AMPK in skeletal muscle function (Herzig and Shaw, 2018; Kjobsted et al., 2018; Thomson, 2018; Wu and Zou, 2020; Drake et al., 2021). But no study has described in detail how AMPK affects mitochondrial dynamics, how it affects skeletal muscle growth and regeneration processes and how AMPK affects various biological processes in skeletal muscle by affecting mitochondria. In this review, we describe the effects of mitochondria on skeletal muscle metabolism. In addition, we summarize the regulatory effects of AMPK on mitochondria and how AMPK regulates skeletal muscle metabolism by regulating mitochondrial dynamics. Finally, we describe the AMPK structure and its main activators. In conclusion, current data suggest that AMPK controls skeletal muscle health and function in part through control of mitochondrial dynamics and muscle metabolism.

## 2 Adenosine monophosphate activated protein kinase and its activators

There are relatively few drugs based on interventions for muscle wasting (Weihrauch and Handschin, 2018). Given that AMPK is involved in multiple pathways in mitochondrial and skeletal muscle metabolism, studies are emerging on AMPK activators that may prove to help regulate mitochondrial health, thereby enhancing cellular metabolism and promoting skeletal muscle health. In this section, we describe the structure of AMPK, the major AMPK activators discovered so far, and some examples of AMPK activators can aid in improving muscle wasting.

### 2.1 Adenosine monophosphate activated protein kinase structure

AMPK is an αβγ heterotrimer that functions as a central regulator of energy homeostasis. It is composed of catalytic *a* subunit (α1 and α2), regulatory β-subunit (β1 and β2) and γ-subunit (γ1, γ2, and γ3) (Stapleton et al., 1996; Yan et al., 2018). These subunits produce 12 different complexes, all of which can be produced in mammalian tissues [Reviewed in (Ross et al., 2016)]. In muscle tissue, AMPK is the core hub of energy metabolism. All combinations of AMPK can be expressed in mammals, but their expression levels differ in different tissues, and α1β2γ1, α2β2γ1, and α2β2γ3 are mainly expressed in skeletal muscle (Birk and Wojtaszewski, 2006). Although there are different heterotrimer subtypes in tissues, their specific roles are still being studied.

### 2.2 Adenosine monophosphate activated protein kinase activation

**FIGURE 1:**
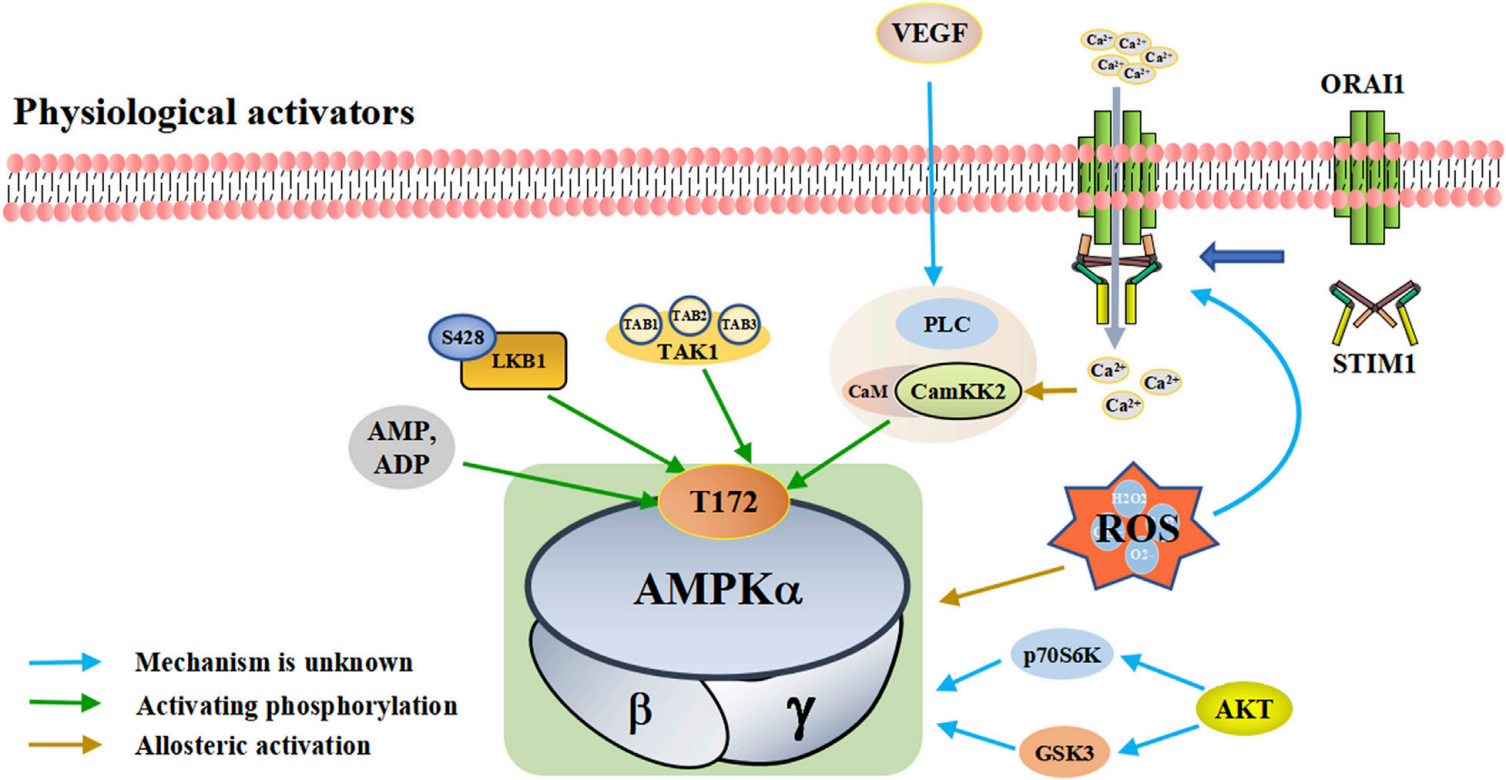
A schematic diagram of the pathways by which physiological activators activate adenosine monophosphate activated protein kinase (AMPK). The physiological activators are substances derived from the host’s own cells or tissues that activate AMPK. Physiological of AMPK include AMP/ADP, upstream kinases (LKB1, CaMKK2, TAK1, and AKT), VEGF and ROS. They are substances produced by the body that activate AMPK in different ways. AMPK is activated when the intracellular AMP/ADP ratio increases. As upstream kinases, LKB1, CaMKK2 and TAK1 can directly activate AMPK. When ROS is increased, excess ROS directly activates AMPK. He can also promote the interaction between STIM1 and Orai1 proximal to the plasma membrane, increasing calcium influx, activating CaMKK2 and subsequently AMPK. STIM1: stromal interaction molecule 1, Orai1: ORAI calcium release-activated calcium modulator 1, Akt: protein kinase B, VEGF: vascular endothelial growth factor, PLC: phospholipase C, LKB1: liver kinase B1, TAK1: TGF-beta-activated kinase 1.

AMPK signal can be activated by “physiological activators” (Table 1) and “pharmacological activators” (Table 2). The physiological activators refer to substances derived from the host’s own cells or tissues, while pharmacological activators refer to substances that do not exist in the host itself, are synthesized or exist in nature. The physiological activators include AMP/ADP, upstream kinases (liver kinase B1 (LKB1), CaMKK2, and TGF-beta-activated kinase 1 (TAK1)) and reactive oxygen species (Figure 1). Many drugs activate AMPK indirectly by mimicking physiological activators or activating physiological activators of AMPK. The pharmacological activators include antidiabetic drugs (metformin, dapagliflozin, empagliflozin), small molecules (AICAR/ZMP, A-769662 and 991, pyrrolopyridines, benzimidazoles, salsalate, PF-249, bempedoic acid, MT63-78, Compound PT1 and so on) and plant-derived extracts (Tanshinone IIA, resveratrol, berberine and quercetin).

**TABLE 1:** The physiological activators of AMPK.

AMPK activators	Name	Effect	References
AMP/ADP	AMP/ADP	Render AMPK better able to be phosphorylated by its upstream kinases, and less able to be dephosphorylated by its phosphatases	Hawley et al. (1996); Sanders et al. (2007)
	AMP/ADP	AMP and ADP bind the γ-subunit to enhance the activation of AMPK, but only AMP allosterically activates AMPK to any great extent	Xiao et al. (2011)
Upstream kinases	LKB1	Phosphorylates Thr-172 on the AMPK *a*-subunit to activate AMPK.	Sakamoto et al. (2005)
	Calcium [Ca2+]/calmodulin [CaM]-dependent protein kinase Cam 2 (CaMKK2)		Anderson et al. (2008); Marcelo et al. (2016); Sabbir et al. (2021)
	Transforming growth factor-β (TGF-β)-activated kinase 1 (TAK1)		Momcilovic et al. (2006); Inokuchi-Shimizu et al. (2014)
	PKD1	Inhibits AMPKα2 activity through phosphorylation of Ser491	Coughlan et al. (2016)
	AKT	Regulates AMPK activity by altering the activities of glycogen synthase kinase 3 and ribosomal protein 70 S6 kinase	Dhani et al. (2020)
	S6k	Inhibits AMPK activity through phosphorylates AMPKα2 Ser491	Dagon et al. (2012)
	PKC	Inhibits AMPK activity through phosphorylates AMPKα1 Ser487	Heathcote et al. (2016)
	PKA	Inhibits AMPK activation through phosphorylates Ser495	Spengler et al. (2020)
Reactive oxygen species	ROS	Can result in the oxidation of cysteine on AMPK *a*- and β-subunits to activate AMPK.	Zmijewski et al. (2010); Cardaci et al. (2012)
		Affect AMPK activity by regulating Ca^2+^-related signaling pathways	Mungai et al. (2011); Roca-Agujetas et al. (2019); Huang et al. (2021)

**TABLE 2:** The pharmacological activators of AMPK.

AMPK activators	Name	Effect	Chemical structure	References
Antidiabetic drugs	Metformin	Inhibits respiratory chain complex I leading to an increase of intracellular AMP or ADP to activate AMPK.	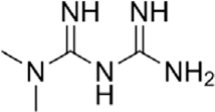	Rena et al. (2017); LaMoia and Shulman (2021)
	Canagliflozin		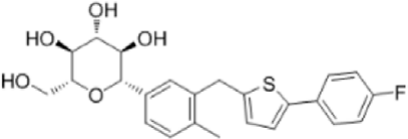	Hawley et al. (2016); Zhou et al. (2020)
	Dapagliflozin	Activate AMPK by increasing p-AMPK/AMPK ratio	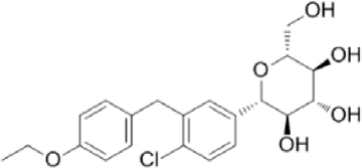	Arab et al. (2021)
	Empagliflozin	Activate AMPK through the LKB1/AMPK signaling pathway and Sesn2-mediated AMPK-mTOR signaling pathway and by slowing down the dephosphorylation rate of DRP1 Ser-637	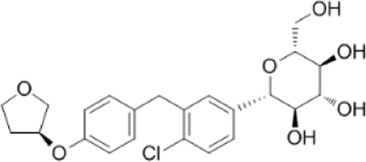	Lu et al. (2020a); Liu et al. (2020); Sun et al. (2020)
Small molecules	5-Aminoimidazole-4-carboxamide ribonucleotide (AICAR)	Phosphorylated by adenosine kinase to produce ZMP, simulating AMP to activate AMPK.	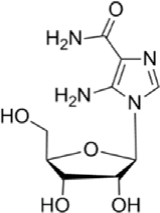	Sabina et al. (1985); Ahmad et al. (2021 )
	5-(5-hydroxy-isoxazol-3-yl)-furan-2-phosphonic acid (C2)	Simulat AMP to activate AMPK.	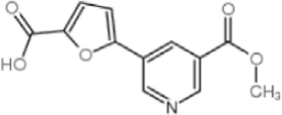	Langendorf et al. (2016)
	2-[2-(4-(trifluoromethyl) phenylamine) thiazol-4-yl] acetic acid (activator-3)		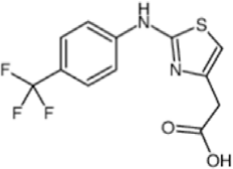	Bung et al. (2018)
	C13	α1-selective AMPK activator	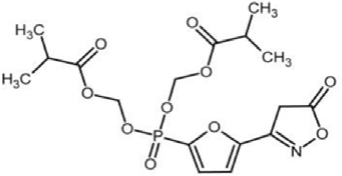	Hunter et al. (2014)
	A-769662	Directly activate AMPK.	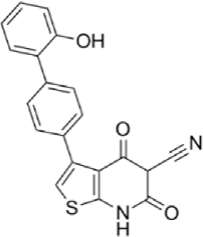	Kopietz et al. (2018)
	991 (ex229)		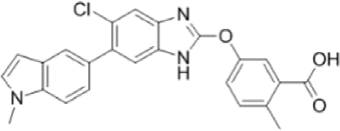	Madhavi et al. (2019)
	Compound PT1		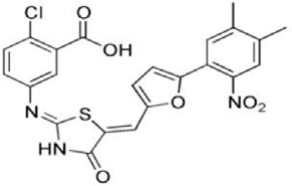	Pang et al. (2008)
	Salsalate	Bind to AMPK β1- and/or β2-subunits to activate AMPK.	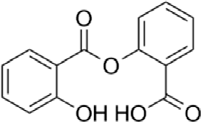	Day et al. (2021)
	PF-249		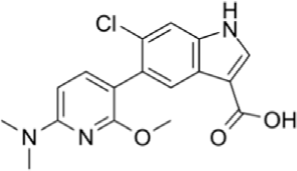	Cokorinos et al. (2017)
	6-chloro-5-[4-(1-hydroxychlorobutyl) phenyl]-1H-indole-3-carboxylic acid (PF-06409,577)		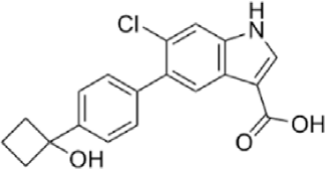	Cameron et al. (2016)
	PF-739		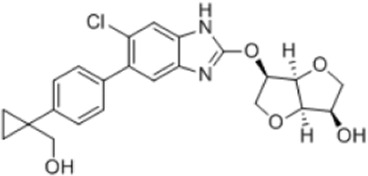	Jorgensen et al. (2021)
	ETC-1002		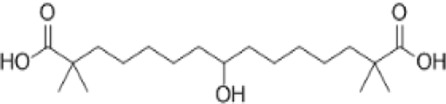	Pinkosky et al. (2016)
	MT63-78		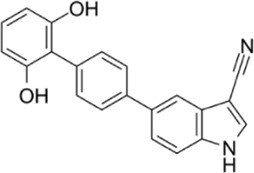	Zadra et al. (2014)
	Compounds SC4		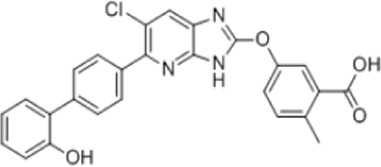	Ngoei et al. (2018)
	MK-8722		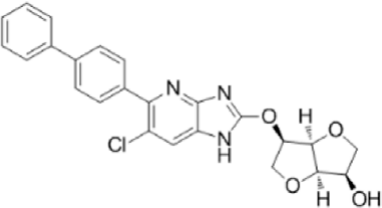	Wang et al. (2021a)
	O304	Inhibits the dephosphorylation of pThr172, thereby prolonging AMPK activation	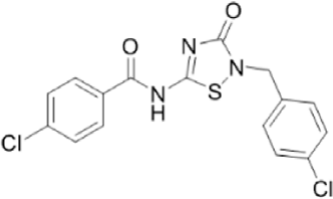	Ericsson et al. (2021)
	Sanguinarine	Phosphorylate the *a*-subunit to activate AMPK.	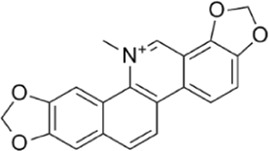	Zhang et al. (2018)
Plant-derived extracts	Tanshinone IIA	Activate AMPK through the AMPK/mTOR-dependent autophagy pathway	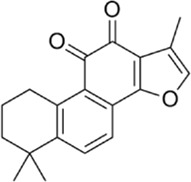	Zhang et al. (2019)
	Flavonoids extracted from mulberry leaves	Improve skeletal muscle mitochondrial function in type 2 diabetes by activating AMPK.		Meng et al. (2020)
	Resveratrol	Increase the phosphorylation/activation of AMPK.	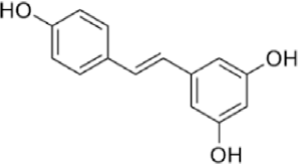	Den Hartogh et al. (2020); Wen et al. (2020)
	Berberine	Mechanism is not clear	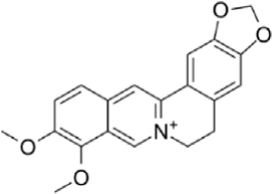	Bijland et al. (2013); Xu et al. (2021)

#### 2.2.1 Physiological activators

##### 2.2.1.1 AMP/ADP

Cell metabolism and various conditions will convert ATP into AMP/ADP. The increase of intracellular AMP/ADP ratio leads to the enhanced phosphorylation of the threonine residues (Thr-172) in the AMPK *a*-subunit and slow down the dephosphorylation rate of Thr-172 (Hawley et al., 1996; Sanders et al., 2007). This enables AMPK activation and promotes ATP production (Gowans et al., 2013). Compared with AMP, ADP has a higher concentration and plays a major controlling role (Coccimiglio and Clarke, 2020). In addition to activating AMPK through Thr-172 phosphorylation, AMP can also bind to the regulatory γ-subunit to activate AMPK (Xiao et al., 2011; Gowans et al., 2013). AMP/ADP is the direct activator of AMPK. Some substances, such as metformin and canagliflozin, can regulate the activity of AMPK by regulating the intracellular levels of AMP/ADP.

##### 2.2.1.2 Upstream kinases

Upstream kinases of AMPK mainly include LKB1, CaMKK2, and TAK1, all of which exert their functions by phosphorylating Thr-172 on the AMPK *a*-subunit (Lou et al., 2021; Zhu et al., 2022). *LKB1* is a tumor suppressor gene that encodes the serine/threonine kinase of calmodulin family expressed in a variety of tissues and is highly conserved in eukaryotes [Reviewed in (Ciccarese et al., 2019)]. LKB1 plays an important role in regulating cell metabolism. LKB1 phosphorylates the AMPK *a*-subunit Thr-172 to activate AMPK (Sakamoto et al., 2005); LKB1 and AMPK together regulate cell growth depending on changes in environmental nutrition (Shackelford and Shaw, 2009).

CaMKK2 (also known as CaMKKβ) belongs to a serine/threonine-specific protein kinase family. When intracellular Ca^2+^ increases due to various reasons, Ca^2+^ binds to CaM to form the Ca^2+^/CaM complex, which activates CaMKK2 phosphorylation (Marcelo et al., 2016; Hedman et al., 2021). Activated CaMKK2 phosphorylates the AMPK *a*-subunit, forming a polyprotein complex composed of Ca^2+^/CAM, CaMKK2, and AMPK, which activates AMPK (Anderson et al., 2008; Marcelo et al., 2016; Sabbir et al., 2021). The CaMKK-AMPK pathway operates as part of signaling pathways downstream of nutrient intake, energy metabolism, adipogenesis, inflammation, and skeletal muscle metabolism (Williams and Sankar, 2019). TAK1 is a serine/threonine protein kinase of the mitogen-activated protein kinase kinase family, which functions by binding to TAB1, TAB2, and TAB3 (Mukhopadhyay and Lee, 2020; Zhu L. et al., 2021). TAK1 can be activated by lipopolysaccharide and TGF-β receptor, tumor necrosis factor-α, toll-like receptor (TLR), interleukin-1 (IL-1), and B-cell receptor (Liu et al., 2018; Jia et al., 2020). The mechanism by which TAK1 controls AMPK remains unclear. It is currently hypothesized that TAK1 regulates AMPK activity through phosphorylation (Momcilovic et al., 2006; Inokuchi-Shimizu et al., 2014).

In addition to the classic AMPK activation by phosphorylation of AMPKα-subunit Thr172, there are other kinases that control AMPK activity through other mechanisms. For example, PKD1 can inhibit AMPKα2 activity through phosphorylation at Ser491 (Coughlan et al., 2016). Protein kinase B (Akt) regulates AMPK activity by altering the activities of glycogen synthase kinase three and ribosomal protein 70 S6 kinase (Dhani et al., 2020). p70S6 kinase phosphorylates AMPKα2 Ser491 to inhibit AMPK activity (Dagon et al., 2012). Protein kinase C (PKC) results in phosphorylation at AMPKα1 Ser487, thereby inhibiting AMPK activity (Heathcote et al., 2016). Vascular endothelial growth factor (VEGF) activates AMPK through CaMKK2 in endothelial cells, but protein kinase A (PKA) inhibits AMPK activation by phosphorylation at Ser495 (Spengler et al., 2020). Further investigation will likely reveal more types of kinases and ensure better understanding of their important roles in AMPK activation and inhibition.

##### 2.2.1.3 Reactive oxygen species

Reactive oxygen species (ROS), including hydrogen peroxide (H2O2), hydroxyl radical (OH−), single oxygen (1O2), and superoxide (O2-), are a group of molecules produced by the mitochondria, peroxisomes, endoplasmic reticulum, cytosol, plasma membrane by NADPH oxidases and so on. (Magnani and Mattevi, 2019; Perillo et al., 2020; Yang and Lian, 2020).

Reductions in nutrition, oxygen, and growth factors, can lead to excessive production of ROS (Zhao et al., 2017). Excessive ROS can result in the oxidation of cysteine residues on AMPK *a*- and β-subunits, which directly activate AMPK (Zmijewski et al., 2010; Cardaci et al., 2012). In addition, ROS can also affect AMPK activity by regulating Ca^2+^-related signaling pathways (Roca-Agujetas et al., 2019). ROS localized proximal to the plasma membrane promotes the interaction between stromal interaction molecule 1 (STIM1) and ORAI calcium release-activated calcium modulator 1 (Orai1), which stimulates Ca^2+^ release and activate the store-operated Ca^2+^ release-activated Ca^2+^ (CRAC), which increases calcium influx, activates CaMKK2, and subsequently activates AMPK (Mungai et al., 2011; Huang et al., 2021). The involvement of ROS in the activation of AMPK signaling pathway may also involve other mechanisms, which need to be further studied and discussed.

#### 2.2.2 Pharmacological activators

##### 2.2.2.1 Antidiabetic drugs

**FIGURE 2:**
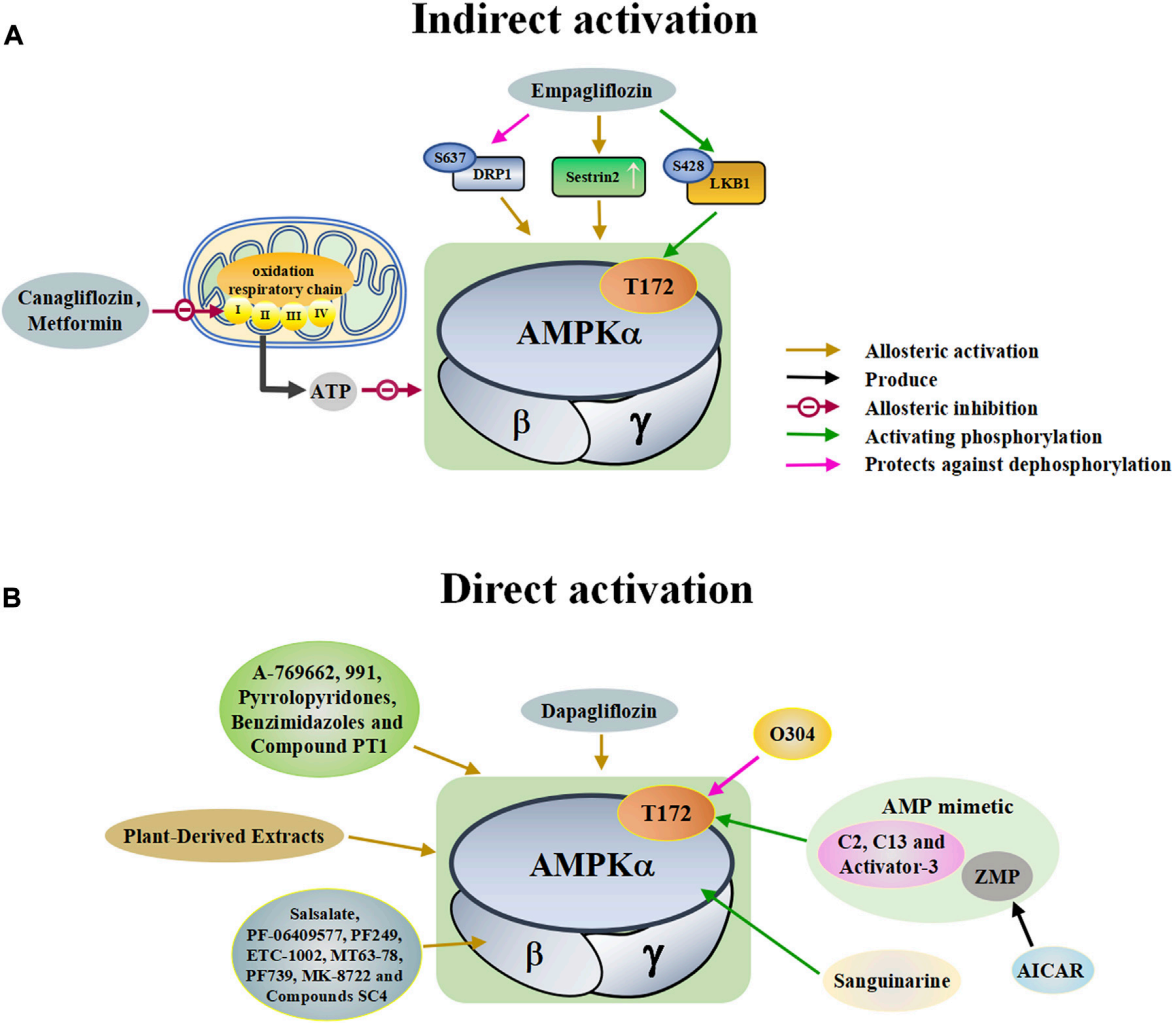
A schematic diagram of the pathways by which pharmacological activators activate adenosine monophosphate activated protein kinase (AMPK). The pharmacological activators refer to substances that do not exist in the host itself but are synthesized artificially or exist in nature that can activate AMPK. **(A)** Indirect activations of AMPK mainly include canagliflozin, metformin and empagliflozin. These substances enter the body and indirectly activate AMPK by controlling molecules that control AMPK. **(B)** Direct activations of AMPK mainly include small molecules, dapagliflozin, plant-derived extracts, O304, sanguinarine, and AICAR. These substances enter the body and directly bind to AMPK to activate AMPK. DRP1: dynamin-related protein1, LKB1: liver kinase B1, ZMP: 5-aminoimidazole-4-carboxamide ribonucleoside monophosphate, C13: Compound 13, ETC-1002: bempedoic acid.

A variety of antidiabetic drugs can directly or indirectly activate AMPK (Al-Ishaq et al., 2019; LaMoia and Shulman, 2021) (Figure 2). Metformin is a first-line drug in the treatment of type II diabetes, one of its effects is to activate AMPK indirectly to affect the treatment of diabetes (Agius et al., 2020; Zhang et al., 2020; Kaneto et al., 2021). Metformin can inhibit the activity of mitochondrial complex I *in vivo*, thus inhibiting the oxidative phosphorylation of mitochondria, increasing ADP/ATP and AMP/ATP ratios in the cells, and activating AMPK indirectly (Rena et al., 2017; LaMoia and Shulman, 2021) (Figure 2A). Canagliflozin, Empagliflozin and Dapagliflozin are all sodium glucose cotransporter 2 (SGLT2) inhibitors, and have shown to activate AMPK in different ways. Canagliflozin inhibits respiratory chain complex I leading to an increase of intracellular AMP or ADP, so as to activate AMPK indirectly (Hawley et al., 2016; Zhou et al., 2020). Empagliflozin can activate AMPK through the LKB1/AMPK signaling pathway and Sesn2-mediated AMPK-mTOR signaling pathway and by slowing down the dephosphorylation rate of DRP1 at serine 637 (Ser-637) (Lu Q. et al., 2020; Liu et al., 2020; Sun et al., 2020). Dapagliflozin can activate AMPK by directly increasing p-AMPK/AMPK ratio (Arab et al., 2021). Although antidiabetic drugs can activate AMPK in multiple ways, their effects after AMPK activation need further investigation.

##### 2.2.2.2 Small molecules

Discoveries of natural compounds and druggable kinases have led to the development of small-molecule compounds that can alter AMPK activity. These small molecule compounds activate AMPK in various ways (Guigas and Viollet, 2016). AICAR is an inosine precursor and an adenosine analogue. After entering the cell, AICAR is phosphorylated by adenosine kinase to produce 5-aminoimidazole-4-carboxamide ribonucleoside monophosphate (ZMP), which is an AMP mimic that activates AMPK (Sabina et al., 1985; Ahmad et al., 2021). Similar AMP analogues include C2 and activator-3 (Langendorf et al., 2016; Bung et al., 2018; Mo et al., 2019). A variety of small molecules can activate AMPK, as shown in Table 2. All of these substances directly activate AMPK (Figure 2B).

##### 2.2.2.3 Plant-derived extracts

Plant-derived extracts have been used in daily therapeutic activities as an effective traditional Chinese medicine, and extracts of many plants have been reported to directly activate AMPK (Francini et al., 2019; Joshi et al., 2019). Tanshinone IIA, flavonoids extracted from mulberry leaves, and resveratrol all act by activating AMPK (Zhang et al., 2019; Den Hartogh et al., 2020; Meng et al., 2020; Vlavcheski et al., 2020; Wen et al., 2020) (Table 2). In addition, many natural products such as berberine and quercetin show great potential in regulating and activating the AMPK pathway (Kjobsted et al., 2018; Wang N. et al., 2021; Xu et al., 2021). These studies suggest that plant-derived extracts can effectively activate the AMPK pathway and provide important information for the development of new drugs for many AMPK-related diseases.

#### 2.2.3 Adenosine monophosphate activated protein kinase activators that act on skeletal muscle

Not all pharmacological activators can act on skeletal muscle due to the specific expression of three AMPK heterotrimers in skeletal muscle (Table 3). Metformin increases AMPK activity in skeletal muscle of subjects with type 2 diabetes (Musi et al., 2002). The small molecules that have been proven to activate AMPK in skeletal muscle include AICAR, 991, PF-739 and MK-8722 (Cokorinos et al., 2017; Myers et al., 2017; Olivier et al., 2018). Plant-derived extracts like flavonoids extracted from mulberry leaves and resveratrol have been shown to activate AMPK in skeletal muscle to regulate skeletal muscle state (Huang et al., 2019; Meng et al., 2020).

**TABLE 3:** Substances that act on AMPK to have a positive effect on the body or cells.

Agonist	Target	Dosage	Species	Function	Mechanism	References
Metformin	Indirect AMPK	20 mg/kg/day, 8 weeks	Mice	Inhibited the NLRP3 Inflammasome	AMPK, mTOR, NLRP3	Yang et al. (2019)
		50 mg/kg/day, 16 weeks	Mice	Reduced hyperglycemia	AMPK, Drp1	Wang et al. (2019)
				Reduced lipid accumulation		
		200 mg/kg, gavage	Rats	Reduced hyperglycemia	AMPK, Stimulate GLP-1 release	Duca et al. (2015)
		350 mg/kg/day, 2 weeks	Mice	Hippocampal neurogenesis	GPD2	DiTacchio et al. (2015)
Canagliflozin	Indirect AMPK	100 mg/kg, gavage	Mice	Inhibited lipid synthesis	AMPK, SGLT2	Hawley et al. (2016)
				Reduced hyperglycemia		
		5 mg/kg/day, 7 days	Rats	Improved kidney function	Ameliorated renal oxidative stress and inflammation	Hasan et al. (2020)
		20–30 mg/kg/day, 8 weeks	Mice	Promoted mitochondrial remodeling of adipocyte	AMPK, Sirt1, Pgc-1α	Yang et al. (2020)
Dapagliflozin	AMPK	5 mg/kg/day, 11 days	Rats	Reduce inflammation	AMPK, activated colonic autophagy and inhibited apoptosis	Arab et al. (2021)
				Protected the intestinal		
		1 mg/kg/day, 9 weeks	Rats	Attenuated hepatic lipid accumulation	AMPK, decreasing lipogenic enzyme	Li et al. (2021b)
				Ameliorated hepatic steatosis		
		1 mg/kg/day, 8 weeks	Rats	Inhibited collagen secretion by fibroblasts	AMPKα, TGF-β, suppressing fibroblast activation	Tian et al. (2021)
				Protected against DCM and myocardial fibrosis		
		3 mg/kg/day, 4 weeks	Rats	Ameliorated pancreatic injury	attenuated oxidative stress, inflammation, apoptosis	Jaikumkao et al. (2021)
				Activated kidney autophagy		
Empagliflozin	Indirect AMPK	3.8 mg/kg/day, 4 weeks	Mice	Alleviated hepatic steatosis	AMPK, elevated autophagy	Li et al. (2020)
		10 mg/kg/day, 8 weeks	Mice	Attenuated hyperuricemia	ABCG2, p-AMPK, p-AKT, p-CREB	Lu et al. (2020b)
		3.8 mg/kg/day, 8 weeks	Mice	Inhibited hepatic gluconeogenesis	AMPK, CREB, GSK3β	Yu et al. (2022)
				Increased glycogen synthesis		
		22 μm	Cells	Attenuated lipotoxicity	CAMKK2, AMPK, antioxidant	Wang et al. (2022c)
				Protected hepatocytes		
AICAR	AMPK	250 μm	Cells	Reduced hepatocyte glucose production	AMPK, ENT1	Logie et al. (2018)
		50 mg/kg/day, 30 days	Mice	Prolonged corneal allograft survival	IB4, VEGF	Jiang et al. (2019)
		150 mg/kg/day, 5 weeks	Mice	Reduced macrophage inflammation	SIRT1	Yang et al. (2012)
		100 mg/kg/day, 5 days	Rats	Protected against acute kidney injury	JAK, STAT, SOCS	Tsogbadrakh et al. (2019)
Activator-3	AMPK	28 nm/32 nm	Cells	Activated AMPK		Bung et al. (2018)
C13	AMPK	10 nm, 2 h	Cells	Protected neuronal cells	Reduced oxidative stress	Mo et al. (2019)
		10 nm, 30 min	Cells	Inhibited gastric epithelial cell apoptosis	Reduced oxidative stress	Zhao et al. (2015)
A-769662	AMPK	100 nm	Cells	Activated AMPK		Kopietz et al. (2018)
		10 mg/kg	Rats	Reduced acute heart inflammation	AMPK, Myd88	Rameshrad et al. (2016)
991 (ex229)	AMPK	20 nm, 2 h	Cells	Induced mitophagy	AMPK, TBK1	Seabright et al. (2020)
				Promoted mitochondrial fission		
		30 nm, 1 h	Muscles	Enhanced glucose uptake induced	AMPK	Bultot et al. (2016)
		5 nM, 45 min		Enhanced contraction in skeletal muscle		
Compound PT1	AMPK	10 nm/20 nm/40 nm	Cells	Activated AMPK	AMPK	Pang et al. (2008)
		100 mg/kg/day, 3 days	Mice	Protect cardiomyocytes after ischemia	Induction of autophagy	Huang et al. (2016)
Salsalate	AMPK	300 mg/kg/day, 7 days	Mice	Reversed metabolic disorders in nonalcoholic fatty liver disease	AMPK, caspase-6	Li et al. (2021a)
		50 mg/kg/day, 5 weeks	Mice	Ameliorated hepatic steatosis	Fetuin-A, AMPK, NFκB	Jung et al. (2013)
		2.5 g/kg, western diet, 6 weeks	Mice	Reduced atherosclerosis	AMPK	Day et al. (2021)
PF-249	AMPK	100 mg/kg	Mice	Reduced hyperglycemia	AMPK	Cokorinos et al. (2017)
PF-739	AMPK	100 mg/kg	Mice	Reduced hyperglycemia	AMPK	
PF-06409,577	AMPK	1 μm	Cells	Inhibited osteosarcoma cell growth	AMPK	Zhu et al. (2021b)
		50μm/100 μm	Cells	Inhibited flavivirus infection	AMPK, modification of cell lipid metabolism	Jimenez de Oya et al. (2018)
ETC-1002	AMPK	100 μm, 5 μL/day, 10 days	Mice	Exerts ameliorative effects in experimental periodontitis	AMPK, NF-κB	Li et al. (2022)
		100 μm	Cells	Regulated immune adipose tissue inflammation	LKB1, AMPK	Filippov et al. (2013)
MK-8722	AMPK	20 μm/50 μm	Cells	Improved glucose homeostasis		Myers et al. (2017)
				Induces cardiac hypertrophy	
				Inhibited carcinoma proliferation, invasion and migration in human pancreatic cancer cells		Wang et al. (2021a)
O304	AMPK	0.5 mg/g, 6 months	Mice	Improved metabolic and cardiac function		Zhu et al. (2022)
				Improved exercise capacity		
Tanshinone IIA	AMPK	1.5 mg/kg/day, 28 days	Rats	Protected against heart failure post-myocardial infarction	AMPKs, mTOR	Zhang et al. (2019)
Flavonoids extracted from mulberry leaves	AMPK	180 mg/kg/day, 7 weeks	Mice	Improved skeletal muscle mitochondrial function	AMPK	Meng et al. (2020)
Resveratrol	AMPK	0.2 g for 0.4% in the diet, 20 weeks	Rats	Prevented sarcopenic obesity	PKA, LKB1, AMPK	Huang et al. (2019)

Although there are many activators that activate AMPK in skeletal muscle, not all of them are useful. AICAR has lost its appeal because of poor selectivity, low potency, inadequate bioavailability, and the potential “off-target” effects in cells [Reviewed in (Visnjic et al., 2021)]. Although 991, PF-739, and MK-8722 can activate AMPK in skeletal muscle and increase glucose uptake of skeletal muscle, their effects on skeletal muscle growth, atrophy, and regeneration are still unclear and need further research. Tanshinone IIA may have potential for the treatment of skeletal muscle wasting, because it can activate AMPK in various ways in different tissues (Yun et al., 2014; Zhang et al., 2014; Li et al., 2018; Zhang et al., 2019). However, whether Tanshinone IIA can activate AMPK in skeletal muscle remains to be further studied.

## 3 The role of mitochondria in myogenesis, regeneration, and muscle atrophy

**FIGURE 3:**
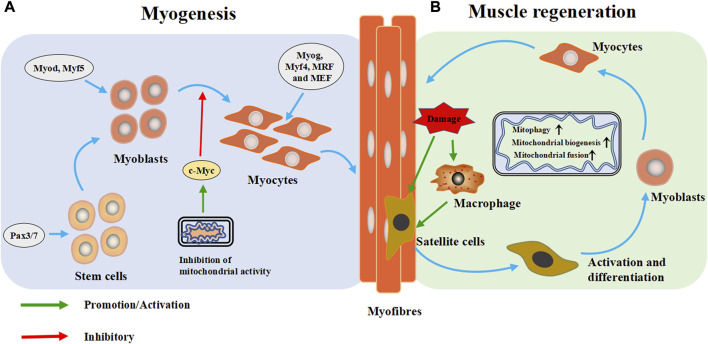
Mitochondria are extensively involved in the metabolic process of skeletal muscle cells. **(A)** Myogenesis: During the embryonic stage, stem cells form muscle progenitor cells under the intervention of regulatory factors and transcription factors, which are then activated and differentiate into myoblasts. Subsequently, the myoblasts exit the cell cycle and differentiate and fuse to form multinucleated myotubes. **(B)** Muscle regeneration: When muscle is injured, skeletal muscle heals itself through a programmed process. During degradation and inflammation, macrophages activate quiescent muscle stem cells (satellite cells) to differentiate into myoblasts, which then differentiate into muscle cells and fuse into myotubes to form muscle fibers and complete skeletal muscle repair. Pax3/Pax7: paired-box 3 and 7 transcription factors, Myod: myoblast determination protein 1, Myf5: myogenic factor 5, Myog: myogenin, Myf4: myogenin, MRF: myogenic regulatory factor, MEF2: myocyte enhancer factor 2.

Skeletal muscle exhibits a remearkable plasiticity, as its morphology and function can exhibit profound adaptations to the demands placed on it (Qaisar et al., 2016). Skeletal muscle tissue is the key determinant of basal metabolic rate and systemic energy metabolism, requiring a large amount of energy to maintain function. Mitochondria in the tissue maximize oxidative phosphorylation through dynamic fusion and fission to maintain cell function (Rahman and Quadrilatero, 2021a). The maintenance of normal mitochondrial function is important for the myogenesis and regeneration of skeletal muscle (Figure 3). Mitochondrial dysfunction can disorder skeletal muscle metabolism, and eventually lead to skeletal muscle atrophy.

### 3.1 The role of mitochondria in skeletal myogenesis

Skeletal myogenesis is the process of forming mature skeletal muscle tissue from precursor cells, which mainly occurs during embryonic and fetal development (Figure 3A). In the embryonic stage, stem cells form muscle progenitor cells under the influence of transcription factors such as the paired-box seven and three transcription factors (Pax7/Pax3), myoblast determination protein 1 (Myod), and myogenic factor 5 (Myf5), which then activate and differentiate into myoblasts. Subsequently, myoblasts exit the cell cycle differentiate, and fuse to form multinucleated myotubes (Bentzinger et al., 2012). As the differentiation progresses, and fuse to form multinucleated myotubes, myogenin (Myog), myogenin (Myf4), myogenic regulatory factor (MRF) and myocyte enhancer factor 2 (MEF2) catalyze subsequent gene expression (Zammit, 2017; Li et al., 2019).

The formation of skeletal muscle is accompanied by the replacement of low-function mitochondria, which eventually leads to the accumulation of high-function mitochondria (Rahman and Quadrilatero, 2021a). Mitochondria can regulate myoblast differentiation by controlling the expression of *c-Myc* gene. When the activity of mitochondria is inhibited, the intracellular expression of c-Myc increases, which will inhibit myogenic differentiation (Seyer et al., 2006). Mitochondrial autophagy plays a role in initiating myogenesis, at least *in vitro* (Rahman and Quadrilatero, 2021a). These studies suggest that normal mitochondrial function plays an important role in the genesis and formation of skeletal muscle.

### 3.2 Mitochondria regulate skeletal muscle regeneration

Skeletal muscle is often injured during sports, and its high regeneration efficiency is important for recovery of its function. In case of muscle injury, skeletal muscle completes self-healing through four progressive steps: degradation, inflammation, regeneration and remodeling (Huard et al., 2002). Regeneration is a programmed process. The process begins with degeneration and inflammation, and during these two steps, macrophages activate quiescent muscle stem cells (satellite cells) to differentiate into myoblasts, which then fuse into myotubes and form muscle fibers to complete skeletal muscle repair (Juban and Chazaud, 2017; Rahman and Quadrilatero, 2021b). Satellite cells are the starting point of skeletal muscle regeneration (Figure 3B).

Mitochondrial biogenesis is necessary during muscle regeneration (Wu et al., 2018; Niu et al., 2021). Under the pressure of differentiation, myoblasts require more energy to maintain cell remodeling; accordingly mitochondria are constantly splitting in cells, and mitochondrial autophagy is markedly increased (Hardy et al., 2016; Bloemberg and Quadrilatero, 2019). Mitochondrial renewal disorder has been repeatedly shown to reduce the differentiation ability of cultured myoblasts and the regeneration ability of skeletal muscle tissue (Baechler et al., 2019; Joseph and Doles, 2021; Qualls et al., 2021). Enhancing mitochondrial biogenesis can improve muscle regeneration (Niu et al., 2021). The combination of mitochondrial biogenesis and fusion promotes energy generation capacity in regenerated skeletal muscle, while inhibition of mitochondrial the protein synthesis inhibits muscle regeneration in injury models (Rahman and Quadrilatero, 2021b). Mitochondrial autophagy is necessary for skeletal muscle regeneration (Rahman and Quadrilatero, 2021a). A previous study showed that after injection of myotoxin, mitochondrial autophagy is inhibited, resulting in delayed regeneration response (Nichenko et al., 2016). Altogether, mitochondria play important roles in skeletal muscle regeneration, but the specific mechanisms remain unclear and needs further study.

### 3.3 The role of mitochondria in muscle atrophy

In chronic diseases, cancer and long-term infections, skeletal muscle can undergo. changes that eventually lead to atrophy (Powers et al., 2020). Muscle atrophy manifests as reductions in muscle mass, fiber cross-sectional area, strength, fatigue resistance, and exercise ability, which may lead to a decline in quality of life and increases in-hospital mortality (Boonyarom and Inui, 2006; Sartori et al., 2021). Skeletal muscle atrophy involves several signal pathways such as ubiquitin proteasome system and autophagy lysosome system (Shen et al., 2019; Wu et al., 2019; Ma et al., 2021; Wang et al., 2022b).

Skeletal muscle atrophy is also related to mitochondrial function, and regulating mitochondrial biogenesis can improve resistance to muscle atrophy (Shen et al., 2020; Jeon and Choung, 2021). When mitochondria are dysfunctional, increased intracellular ROS level activates apoptosis-related signaling pathways and the degradation of many proteins (Theilen et al., 2017).

**FIGURE 4:**
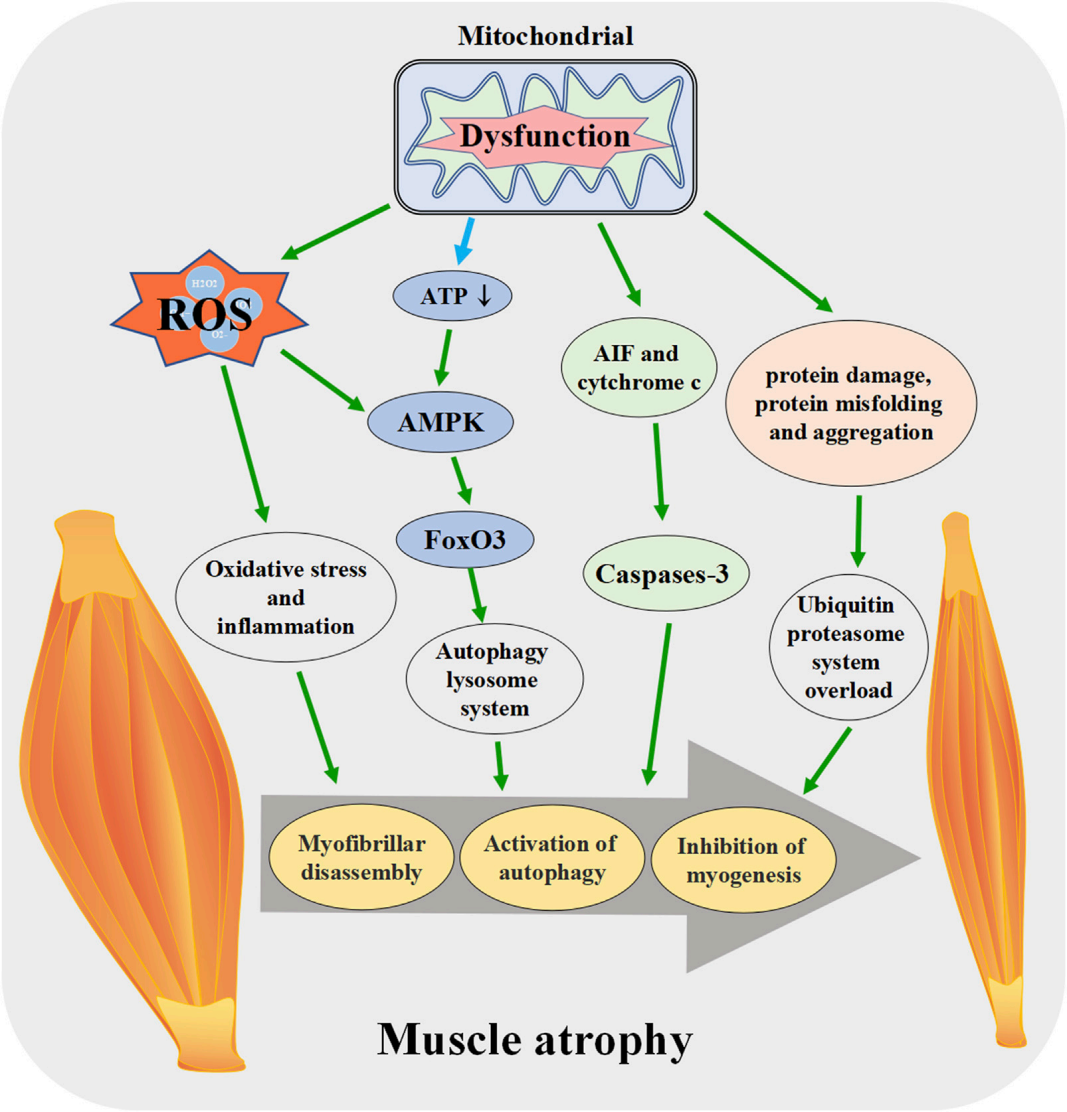
Mitochondrial dysfunction promotes the skeletal muscle atrophy. When mitochondrial function is disrupted, ROS production increases, ATP synthesis decreases and other pathways lead to the activation of apoptosis pathway in muscle tissue, enhanced protein degradation, increased autophagy, muscle fiber breakdown, and eventually induce skeletal muscle atrophy. ROS: reactive oxygen species, AIF: apoptosis-inducing factor, FoxO3: forkhead box O 3.

Mitochondrial dysfunction releases mitochondrial protein apoptosis-inducing factor (AIF) and cytochrome c into the cytosol, which leads to the activation of caspase-3, promotes actin/myosin decomposition, and induces myonuclear cell apoptosis (Delavallee et al., 2020). The proteolytic system activated by AIF and cytochrome c may play an important role in the entire process of muscle atrophy in synergy with other signal transduction effectors [Reviewed in (Hyatt et al., 2019)]. Mitochondrial fission during mitochondrial dysfunction disrupts intracellular energy homeostasis, reduces ATP production, increases the relative concentration of AMP and activates AMPK. AMPK increases the expression of autophagy-specific gene proteins (ATGs) by activating the transcription factor forkhead box O 3 (FoxO3), which leads to the initiation of autophagy and ultimately to skeletal muscle atrophy (Sanchez et al., 2012; Cannavino et al., 2015). The above research results indicate that mitochondrial dysfunction can lead to muscle atrophy in various ways, and regulating mitochondrial function plays a role in resisting muscle atrophy (Figure 4).

## 4 Effects of adenosine monophosphate activated protein kinase on mitochondrial dynamics and skeletal muscle

### 4.1 Effects of adenosine monophosphate activated protein kinase on mitochondrial biogenesis

**FIGURE 5:**
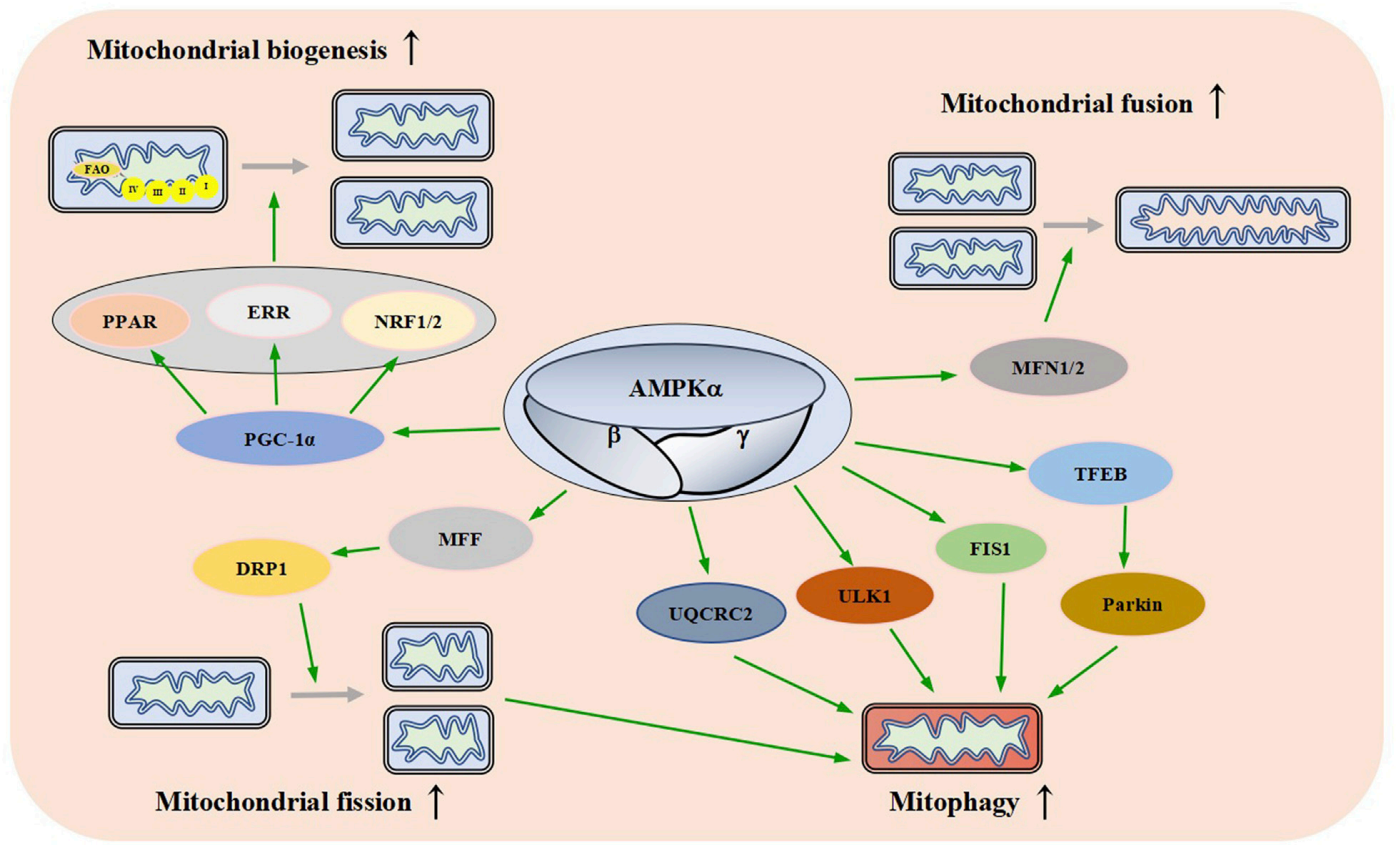
Effects of AMPK on Mitochondrial Dynamics. AMPK promotes mitochondrial biogenesis, fusion, fission, and autophagy through different signaling pathways. FAO: fatty acid oxidation, PPAR: peroxisome proliferator-activated receptor, ERR: estrogen-related receptor, NRF1/2: nuclear respiratory factor 1/nuclear respiratory factor 2, PGC-1a: peroxisome proliferator-activated receptor gamma coactivator-1 alpha, MFN1/2: mitofusin 1/2, MFF: mitochondrial fission factor, DRP1: dynamin-related protein1, UQCRC2: ubiquinol-cytochrome c reductase core protein 2, Ulk1: autophagy activating kinase 1, FIS1: mitochondrial fission one protein, TFEB: transcriptional activity of transcription factor EB.

Mitochondrial biogenesis can be considered as the growth and division of early-stage mitochondria (Jornayvaz and Shulman, 2010). It is affected by the energy demand of cells. Mitochondrial biogenesis-related pathways are activated in response to increased energy consumption conditions such as exercise, hypothermia, oxidative stress, and cell division and differentiation, resulting in changes in the number, size, and mass of mitochondria (Jornayvaz and Shulman, 2010; Popov, 2020). PGC-1α is a member of the transcriptional coactivator family. It is also considered the core molecule in mitochondrial biogenesis (Figure 5). PGC-1α interacts with transcription factors such as peroxisome proliferator-activated receptor (PPAR), estrogen-related receptor (ERR) family, and nuclear respiratory factor 1/nuclear respiratory factor 2 (NRF1/2) to activate almost all mitochondrial biogenesis pathways, including respiratory chain and fatty acid oxidation (FAO) genes, which increases the number of mitochondria and strengthens respiratory capacity (Scarpulla et al., 2012; Zhou et al., 2021).

When AMPK is activated by various stimuli, it induces the expression of PGC-1α by phosphorylation, resulting in an increased activity and thereby promoting mitochondrial biogenesis (Sun et al., 2022). These data suggest that AMPK plays an important role in mitochondrial biogenesis.

### 4.2 Role of adenosine monophosphate activated protein kinase in mitochondrial fusion and fission

Mitochondria are highly dynamic organelles that continuously fuse and divide in different states of cell cycle; mitochondrial fusion and division play an important role in maintaining mitochondrial homeostasis and cellular function (Lee and Yoon, 2016; Sabouny and Shutt, 2020). Fusion helps mitigate stress by mixing the contents of partially damaged mitochondria as a form of complementation. Fission is necessary for the creation of new mitochondria, it provides the raw material for new mitochondria and also contributes to quality control by the removal of damaged mitochondria and facilitates apoptosis (Adebayo et al., 2021). Mammalian mitochondrial fusion is mediated by mitofusin 1/2 (MFN1/2) and OPA1 (Mishra et al., 2014; Gao and Hu, 2021). Mitochondrial division is mainly mediated by mitochondrial fission factor (MFF), dynamin-related protein1 (DRP1), human mitochondrial dynamics proteins 49/51 (MID49/51) and mitochondrial fission one protein (FIS1) (Otera et al., 2016; Kalia et al., 2018; Hu et al., 2021; Konig et al., 2021). AMPKα1 interacts with and phosphorylates MFN2, the adenosine derivative cordycepin induces upregulation of MFN2 in cardiomyocytes in an AMPK-dependent manner to promote mitochondrial fusion (Yu et al., 2021).

Direct pharmacological activation of AMPK can induce mitochondrial fission (Toyama et al., 2016; Trewin et al., 2018). Sustained energy stress activates AMPK, which binds to and phosphorylates MFF, resulting in mitochondrial translocation of DRP1 (Zhang and Lin, 2016; Zheng et al., 2018). The dynamic regulation of mitochondrial fusion and fission mediated by multiple pathways ensures the stability of mitochondrial function (Figure 5).

### 4.3 The role of adenosine monophosphate activated protein kinase in mitochondrial autophagy

Mitochondrial autophagy is a catabolic process that helps maintain mitochondrial quality control by transporting damaged mitochondria to the lysosome for the degradation (Pickles et al., 2018). Mitochondrial autophagy is a protective mechanism of cells, which can reduce intracellular ROS, mtDNA damage, and the accumulation of aging or damaged mitochondria (Williams and Ding, 2018; Onishi et al., 2021).

AMPK plays an important role in autophagy (Herzig and Shaw, 2018). A lot of research supports this idea. In a mouse model of leukemia, AMPK activation upregulates FIS1-mediated mitochondrial autophagy to promote the degradation of mitochondria subjected to stress and maintains the health of the mitochondrial network (Pei et al., 2018). Meanwhile, a study has found that AMPK indirectly up-regulates the expression of ubiquinol-cytochrome c reductase core protein 2 (UQCRC2) to enhance mitochondrial autophagy (Lu et al., 2021). Laker and others found that AMPK phosphorylates autophagy activating kinase 1 (Ulk1) and plays a role in mitochondrial autophagy induced by acute exercise in mouse skeletal muscle (Laker et al., 2017). A study has found that AMPK activation causes transcriptional activity of transcription factor EB (TFEB) transcription and induces Parkin-dependent mitochondrial autophagy to lessen oxidative stress, thereby enhancing mitochondrial function (Cao et al., 2020). There is also one study that has found AMPK promotes fission by phosphorylating MFF, thereby promoting autophagic clearance of damaged mitochondria (Toyama et al., 2016). These results suggest that AMPK links energy metabolism to mitochondrial autophagy through a variety of signaling pathways (Figure 5).

### 4.4 Adenosine monophosphate activated protein kinase influences skeletal muscle protein metabolism *via* mitochondrial function

The mass of adult individual skeletal muscle is mainly determined by the relative rates of the protein synthesis and degradation. When the protein synthesis efficacy is greater than protein degradation efficacy, the mass and volume of skeletal muscle increase. When protein degradation rate is greater than the protein synthesis efficacy, it causes skeletal muscle atrophy (Jaiswal et al., 2019; Romanello and Sandri, 2021).

AMPK can regulate the balance of the protein synthesis and degradation in skeletal muscle. Under healthy conditions, AMPK inhibits the protein synthesis, but under conditions of mitochondrial dysfunction, activation of AMPK might help preserve muscle the protein synthesis by promoting the synthesis of healthy mitochondria. Under physiological conditions, AMPK activation inhibits the protein synthesis and promotes protein breakdown to impair muscle hypertrophy through a variety of pathways (Thomson and Gordon, 2005; Gordon et al., 2008). AMPK inhibits the protein synthesis by inhibiting the activities of mechanistic target of rapamycin, complex 1 (mTORC1) and eukaryotic elongation factor 2 (eEF2) (Thomson, 2018). AMPK can increase FoxO activity through the NAD^+^/sirtuin one pathway to promote protein degradation (Canto et al., 2009). AMPK phosphorylation is negatively correlated with the growth of skeletal muscle, and overexpression of CaMKK2 inhibits the proliferation and differentiation of C2C12 myoblasts by activating AMPK (Ye et al., 2016). However, under pathological conditions, activation of AMPK promotes muscle regeneration and ameliorates muscle atrophy by promoting mitochondrial metabolic activity through different pathways. Activation of AMPK enhances PGC-1α transcription and its coactivator activity, stimulates mitochondrial biogenesis, and promotes muscle regeneration (Quattrocelli et al., 2022). Activation of AMPK enhances satellite-cell proliferation and promotes myogenic differentiation of satellite cells in regenerated muscle (Fu et al., 2016). Under normal conditions, in which sufficient energy is available to support the protein synthesis, the activation of AMPK would operate to slow this rate. In contrast, in conditions in which energy supply is insufficient to support the normal rate of the protein synthesis, such as with mitochondrial dysfunction, AMPK can help to promote the protein synthesis. In this way, AMPK can both limit and enhance muscle growth and regeneration.

In view of the positive and negative regulatory roles of AMPK in skeletal muscle metabolism, its effect on the biological process of skeletal muscle needs to be further investigated.

## 5 Adenosine monophosphate activated protein kinase activators can improve muscle disease status

Many studies have shown that activation of AMPK can effectively prevent or improve muscle disease status.

Qiangji Jianli decoction has been shown to improve muscle atrophy in myasthenia gravis by promoting mitochondrial biosynthesis and restoring muscle energy supply through activation of the AMPK/PGC-1α pathway (Jiao et al., 2020). Resveratrol prevents muscle atrophy caused by a high-fat diet in older adult rats by reversing mitochondrial dysfunction and oxidative stress through the PKA/LKB1/AMPK pathway (Huang et al., 2019). AMPK phosphorylation activates PGC-1α, up-regulates nuclear factor erythroid-derived 2-related factor 1 (Nrf1) expression, enhances energy metabolism, and inhibits skeletal muscle cell apoptosis (Jiang et al., 2020). AMPK can also reduce apoptosis by inhibiting mTOR signaling, increase autophagy by ULK1, and reduce fibrosis by inhibiting transforming growth factor-beta (TGF-beta) signaling (Timm and Tyler, 2020). Various other AMPK activators have shown various beneficial effects in mouse, rat, and cell studies, as shown in Table 3. AMPK activators have been noted and used in the treatment of muscle-related diseases, and as research continues, these activators may be added to the list of therapeutics for muscle-related diseases.

## 6 Perspectives

In recent years, several studies have confirmed that AMPK is the central hub of intracellular energy metabolism regulation. Although AMPK is not the only biological molecule regulating mitochondrial biogenesis, fusion, fission, and autophagy, it is considered to be a core molecule for the maintenance of mitochondrial homeostasis. Owing to the high energy demand of skeletal muscle tissue, mitochondria are important cellular organelles in skeletal muscle tissue. The metabolism of mitochondria affects the development, atrophy, and regeneration of skeletal muscle. Therefore, based on the relationship among AMPK, mitochondria, and skeletal muscle, it can be considered that AMPK can regulate the state of skeletal muscle by regulating mitochondria. Although many studies have shown that drugs can regulate the biological process of mitochondria by first regulating AMPK activity, followed by regulating the metabolism of skeletal muscle, the specific mechanism remains unclear, and several issues need to be addressed. Given that the subtypes of AMPK expressed in different tissues are different, it remains to be seen whether we can develop skeletal muscle-specific drugs that can regulate AMPK activity and improve skeletal muscle metabolism, thereby aiding in disease treatment.
